# Real-World Dermatologic Adverse Events of CAR T-Cell Therapy: A Decade-Wide Disproportionality Analysis of the FDA Adverse Event Reporting System

**DOI:** 10.3390/cancers18132128

**Published:** 2026-06-30

**Authors:** Manideepa Maji, Saikat Mandal, Arkadeep Dhali, Ashish Sharma

**Affiliations:** 1 Hull York Medical School, University of Hull, Hull HU6 7RX, UK; manideepa.maji@hyms.ac.uk; 2 Haematology, Hull University Teaching Hospitals NHS Trust, Hull HU16 5JQ, UK; 3 Translational Medical Sciences, School of Medicine, University of Nottingham, Nottingham NG7 2UH, UK; saikat.mandal@nottingham.ac.uk; 4 NIHR Nottingham Biomedical Research Centre, Nottingham University Hospitals NHS Trust and the University of Nottingham, Nottingham NG7 2UH, UK; 5 Sheffield Teaching Hospitals NHS Trust, Sheffield S5 7AU, UK; 6 School of Medicine, Dentistry and Biomedical Sciences, Queen’s University Belfast, Belfast BT9 7BL, UK; 7 Yale New Haven Hospital, New Haven, CT 06510, USA; ashish.sharma@ynhh.org

**Keywords:** CAR T-cell therapy, dermatologic adverse events, FAERS, pharmacovigilance, severe cutaneous adverse reactions, cytokine release syndrome

## Abstract

**Background:** Chimeric antigen receptor (CAR) T-cell therapy has transformed haematological-malignancy care, but its dermatologic safety profile remains incompletely characterised across products and indications. **Methods:** We analysed 8,431,841 deduplicated FDA Adverse Event Reporting System reports from 2016 Q3 to 2026 Q1. Seven approved CAR T-cell products were identified. The primary outcome was any dermatologic adverse event, defined using the MedDRA Skin and subcutaneous tissue disorders system organ class. Secondary outcomes included broad severe cutaneous adverse reactions, narrow Stevens-Johnson syndrome/toxic epidermal necrolysis, and 14 phenotype-specific categories. Multivariable models adjusted for demographics, polypharmacy, cancer, immune checkpoint inhibitor exposure, lymphodepleting chemotherapy and cytokine release syndrome. Additional sensitivity analyses evaluated HSCT/GVHD co-reporting proxies, infection and cytopenia/bleeding proxies, severe-event clinical characteristics, comparator robustness and multiplicity correction. **Results:** Dermatologic adverse events were identified in 996,654 reports, including 425 CAR-T-associated cases. CAR T-cell exposure was associated with reduced adjusted reporting odds for the primary outcome (adjusted odds ratio 0.13, 95% confidence interval 0.09–0.20) and broad severe cutaneous adverse reactions (0.35, 0.23–0.52). The primary SKIN_ANY reduced-reporting pattern was consistent across all-FAERS, haematological-malignancy and active haematology-oncology comparators. HSCT/GVHD proxy co-reporting was uncommon and did not materially alter estimates. Severe dermatologic reports frequently co-mentioned CRS and serious outcomes. The tisagenlecleucel vascular cutaneous signal was nominally significant but attenuated after excluding infection-attributable and cytopenia/bleeding-proxy reports. **Conclusions:** Within spontaneous reporting systems, CAR T-cell therapy showed reduced relative reporting of dermatologic adverse events across broad, phenotype-specific and product-level analyses. These results should be interpreted as differences in reporting behaviour, not as evidence of reduced true clinical incidence or lower patient-level risk. Early severe cutaneous reports frequently overlapped with cytokine release syndrome, while infection, cytopenia/bleeding proxies and supportive-care drugs were important alternative explanations for selected cutaneous signals.

## Simple Summary

CAR T-cell therapy is an important treatment for some blood cancers, but skin-related side effects after treatment are not well understood. We analysed more than 8.4 million reports from the FDA Adverse Event Reporting System to examine skin adverse-event reporting across seven approved CAR T-cell products. Overall, CAR T-cell therapy was not associated with increased reporting of skin adverse events compared with other reports in the database. Severe skin events were uncommon, but when reported, they often occurred early after treatment and overlapped with cytokine release syndrome, infection, low blood counts, bleeding-related features, or supportive-care medicines. One small vascular skin lesion associated with tisagenlecleucel was observed, but this became weaker after accounting for infection and cytopenia/bleeding-related reports. These findings should not be interpreted as showing that CAR T-cell therapy reduces the true clinical risk of skin toxicity, because spontaneous reporting databases measure reporting patterns rather than incidence. Clinically, a new severe rash or vascular skin changes after CAR T-cell therapy should prompt careful assessment for cytokine release syndrome, infection, cytopenias or bleeding, supportive care drug reactions, and true immune-mediated severe cutaneous adverse reactions.

## 1. Introduction

Chimeric antigen receptor (CAR) T-cell therapy has become an established treatment modality for relapsed or refractory haematological malignancies, particularly through CD19-directed products for B-cell malignancies and B-cell maturation antigen (BCMA) directed products for multiple myeloma [1]. As clinical use expands across products and indications, the safety profile of CAR T-cell therapy requires continued refinement beyond the well-characterised syndromes of cytokine release syndrome (CRS), immune effector cell-associated neurotoxicity syndrome (ICANS), cytopenias and infection [2,3]. Dermatologic adverse events are clinically relevant in this setting because skin findings may reflect immune-mediated toxicity, cytokine-driven inflammation, infection, thrombocytopenia-related bleeding, reactions to antimicrobial or supportive-care drugs, or manifestations of systemic CAR T-cell toxicity.

Compared with CRS and ICANS, the cutaneous safety profile of CAR T-cell therapy remains less well defined. Early clinical reports and subsequent dermatology-focused reviews have described maculopapular eruptions, pruritus, erythema, purpura, bullous reactions, vascular lesions and rare severe cutaneous adverse reactions, including SJS/TEN-like presentations [4,5]. Interpretation is difficult because patients receiving CAR T-cell therapy are frequently exposed to lymphodepleting chemotherapy, antimicrobial prophylaxis or treatment, transfusion support and multiple concomitant medications. In routine practice, timing relative to CRS, cytopenias, infection and concomitant drugs is central to clinical attribution.

Existing evidence remains limited by small cohorts, heterogeneous dermatologic definitions and incomplete product-level or indication-level evaluation. A prior FAERS pharmacovigilance analysis focused mainly on axicabtagene ciloleucel and tisagenlecleucel and reported increased reporting of severe cutaneous eruptions and vascular cutaneous events [6]. More recently, a single-centre cohort provided systematic clinical data but still highlighted small sample size, possible alternative aetiologies and the need for prospective dermatologic assessment [7]. Larger, product-aware and indication-aware analyses are therefore required to place individual cutaneous signals within the broader CAR T-cell treatment context.

There are biologically plausible reasons why dermatologic reporting after CAR T-cell therapy may differ from that observed with other immune-oncology treatments. Lymphodepletion is an integral part of the CAR T-cell treatment cycle and acts by depleting and modulating endogenous lymphocytes, preparing the host microenvironment, and supporting CAR T-cell expansion and persistence [8]. This may reduce some T-cell-mediated inflammatory skin phenotypes. Conversely, early CRS may generate systemic cytokine activation and inflammatory skin findings that can mimic classical drug eruptions. Cytopenias and endothelial activation may also contribute to purpuric or vascular cutaneous presentations.

We therefore analysed deduplicated reports from FAERS, a spontaneous reporting database used for post-marketing safety surveillance of marketed drugs and therapeutic biologics [9]. We evaluated dermatologic adverse-event reporting across seven approved CAR T-cell products from 2016 Q3 to 2026 Q1, with emphasis on broad dermatologic reporting, severe cutaneous adverse reactions, phenotype-specific categories, product-level and indication-level patterns, temporal stability, time-to-onset and overlap with CRS.

## 2. Methods

This retrospective pharmacovigilance study used quarterly FAERS extracts from 2016 Q3 to 2026 Q1 and followed the READUS-PV reporting framework for disproportionality analyses using individual case safety reports [10]. Demographics, drug, reaction, indication, therapy date, and outcome tables were linked. Reports were deduplicated by retaining the most recent case version within composite report keys, using principles consistent with standardised spontaneous-report deduplication approaches [11], yielding 8,431,841 unique reports. The study used publicly available, de-identified data and did not require institutional review board approval.

Seven approved CAR T-cell products were identified using composite regular expressions applied to verbatim drug names and active ingredients. CD19-directed products included tisagenlecleucel, axicabtagene ciloleucel, brexucabtagene autoleucel, lisocabtagene maraleucel and obecabtagene autoleucel. BCMA-directed products included idecabtagene vicleucel and ciltacabtagene autoleucel. Product-level and class-level exposure flags were generated for primary-suspect and any-role reporting.

The primary outcome was any dermatologic adverse event (SKIN_ANY), defined by the MedDRA Skin and subcutaneous tissue disorders system organ class and supported by phenotype-specific regular expressions (Supplementary Table S8). Secondary outcomes included a broad severe cutaneous adverse reaction (SCAR) outcome and a narrow SJS/TEN sensitivity outcome. A 14-category dermatologic adverse-event panel, adapted from Storgard et al. [6], included bullous dermatosis, eczematous dermatitis, psoriasiform reactions, rash, severe cutaneous eruption, toxic eruption, urticaria/angioedema, hair changes, hyperhidrosis, pruritus, skin lesions, vascular cutaneous events, wound/ulcer, and other skin changes (Supplementary Table S8). Category-level exclusion expressions were applied to remove non-cutaneous or off-target preferred terms.

Briefly, the phenotype-specific categories grouped MedDRA Preferred Terms into clinically interpretable patterns: bullous and severe-cutaneous-eruption categories captured blistering and SCAR-spectrum terms; toxic eruption captured drug-eruption terminology; vascular cutaneous captured petechiae, purpura, ecchymosis, skin haemorrhage and cutaneous vasculitis terms; wound/ulcer captured ulcerative and wound-related terms; and chronic or inflammatory categories captured pruritus, eczematous, psoriasiform, urticarial/angioedema, hair-change and hyperhidrosis terms. The complete term logic, including exclusions of off-target non-cutaneous terms, is provided in Supplementary Table S8 and the Supplementary Methods.

Class-level disproportionality was assessed using RORs, proportional reporting ratios, information component and empirical Bayes geometric means. A positive signal required concordance across all prespecified algorithms. Multivariable logistic regression adjusted for age, sex, reporting region, polypharmacy, cancer, immune checkpoint inhibitor exposure, lymphodepleting chemotherapy and CRS co-mention. Prentice-Pyke case-control subsampling was used for rare outcomes [12]. Strong SJS/TEN culprit drugs were defined as lamotrigine, trimethoprim-sulfamethoxazole, phenytoin, allopurinol and carbamazepine, informed by recent SJS/TEN pharmacovigilance work [13]. Additive interaction was assessed using the relative excess risk due to interaction, the attributable proportion, and the synergy index [14,15].

Product-level disproportionality was estimated for each CAR T-cell product in the primary-suspect role against the all-FAERS comparator. Indication-stratified models were fitted within CAR-T-exposed reports using diffuse large B-cell lymphoma (DLBCL) as the reference. Time-to-onset was calculated from the therapy start date to the event date, where available, and was restricted to 1–180 days. Sensitivity analyses excluded strong SJS/TEN culprit drugs, concomitant immune checkpoint inhibitors and events occurring within the first 14 days after infusion. External validation was performed by replicating the analytic frame and by applying analogous class-level analyses in Canada Vigilance [16].

Additional sensitivity analyses were performed to improve clinical interpretability and address potential confounding. Prior haematopoietic stem-cell transplantation (HSCT), graft-versus-host disease (GVHD) and transplant-associated endothelial injury were evaluated using conservative co-reporting proxy terms because prior transplant history is not reliably captured in FAERS. Infection-attributable cutaneous events and cytopenia/bleeding proxies were also defined from the reaction and drug fields. Severe dermatologic reports were characterised by serious outcomes, CRS, infection, cytopenia, culprit-drug co-exposure and co-reported management agents. Comparator robustness was assessed across all-FAERS, haematological-malignancy indication and active haematology-oncology therapy comparators. Multiplicity was assessed using Benjamini–Hochberg false discovery rate correction for the 14-category and product-outcome analyses (Supplementary Tables S9–S13).

## 3. Results

The deduplicated FAERS cohort comprised 8,431,841 reports, including 19,200 CAR-T-exposed reports. CD19-directed products accounted for most CAR-T-exposed reports, while BCMA-directed products accounted for 5444 reports. CRS was co-mentioned in 10,560 CAR-T-exposed reports (55.0%), and 1442 reports (7.5%) included co-exposure to a strong SJS/TEN culprit drug, most commonly trimethoprim-sulfamethoxazole or allopurinol (Table 1).

The primary SKIN_ANY outcome was identified in 996,654 reports (11.8%), including 425 CAR-T-associated cases. Multivariable analysis showed reduced adjusted reporting odds for CAR T-cell exposure for SKIN_ANY (aOR 0.13, 95% CI 0.09–0.20). Broad SCAR was identified in 30,071 reports, including 38 CAR-T-associated cases. CAR T-cell exposure was also associated with reduced adjusted broad SCAR reporting (aOR 0.35, 95% CI 0.23–0.52; *p* < 0.001). Lymphodepleting chemotherapy was associated with lower SCAR reporting odds (aOR 0.54), whereas CRS co-mention was associated with higher SCAR reporting odds (aOR 3.70; Table 2).

Additional HSCT/GVHD sensitivity analyses did not support prior HSCT/GVHD co-reporting as an explanation for the main findings. HSCT/GVHD proxy terms were present in 101 of 19,200 CAR-T reports (0.5%) and 9 of 425 CAR-T-associated SKIN_ANY reports (2.1%). HSCT/GVHD co-reporting was absent from CAR-T-associated broad SCAR, narrow SJS/TEN, vascular cutaneous reports and tisagenlecleucel-associated vascular cutaneous reports, although one broad SCAR report and one vascular cutaneous report included a transplant-associated endothelial proxy term. Adding the HSCT/GVHD proxy did not materially change the CAR-T adjusted odds ratios for SKIN_ANY, broad SCAR, or vascular cutaneous events (Supplementary Table S9).

**Figure 1:**
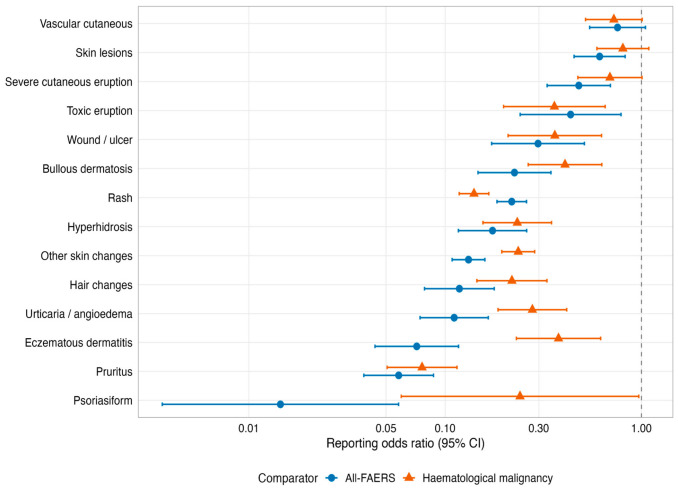
Dermatologic adverse-event reporting in CAR T-cell-exposed reports across 14 phenotype-specific categories, compared with all-FAERS and haematological-malignancy comparators. Points show reporting odds ratios from disproportionality analyses, and horizontal bars show 95% confidence intervals. Estimates below 1 indicate lower relative reporting in CAR-T-exposed reports. Multiple-testing adjustment for the 14-category family is provided in Supplementary Table S13. ROR = reporting odds ratio; CI = confidence interval.

Across the 14-category dermatologic adverse-event panel, RORs were below unity for all categories and statistically reduced in 13 of 14 categories. The lowest estimates were observed for chronic or T-cell-mediated dermatoses, including psoriasiform events (ROR 0.014), pruritus (0.058), eczematous dermatitis (0.072), urticaria/angioedema (0.111) and hair changes (0.118). The weakest reductions occurred in acute drug eruption or vascular categories, including toxic eruption (0.436), severe cutaneous eruption (0.479), skin lesions (0.612), and vascular cutaneous events (0.755; 95% CI 0.545–1.048). Findings were directionally consistent when a haematological-malignancy comparator was used (Figure 1).

**Table 1:** Cohort description.

Characteristic	Value
Total deduplicated FAERS reports	8,431,841
Time window	2016 Q3–2026 Q1
CAR T-cell exposure, any role	19,200 (0.23%)
CAR T-cell exposure, primary suspect	16,888 (0.20%)
CD19-targeting products	13,774
BCMA-targeting products	5444
Median age in CAR-T cohort, years (IQR)	62 (50–69)
Male sex in CAR-T cohort	9180 (47.8%)
Concomitant ICI exposure in CAR-T cohort	76 (0.4%)
Lymphodepleting chemotherapy in CAR-T cohort	5376 (28.0%)
CRS co-mention in CAR-T cohort	10,560 (55.0%)
Strong SJS/TEN culprit co-exposure in CAR-T cohort	1442 (7.5%)
SKIN_ANY events overall	996,654 (11.8%)
CAR-T-associated SKIN_ANY events	425
Broad SCAR events overall	30,071 (0.36%)
CAR-T-associated broad SCAR events	38
Narrow SJS/TEN events overall	12,310 (0.15%)
CAR-T-associated narrow SJS/TEN events	29

ICI = immune checkpoint inhibitor; CRS = cytokine release syndrome; SCAR = severe cutaneous adverse reactions; SJS/TEN = Stevens–Johnson syndrome/toxic epidermal necrolysis. CAR T-cell exposure in Table 1 is shown for any reporter-assigned role unless otherwise specified. Product-level analyses in Table 3 use the primary-suspect role only. CD19- and BCMA-targeting categories may not be mutually exclusive because some reports contained more than one CAR T-cell product or target flag.

**Table 2:** Main adjusted and disproportionality results. Adjusted odds ratios were estimated using multivariable logistic regression; reporting odds ratios were estimated from 2 × 2 disproportionality tables. Estimates are shown with 95% confidence intervals. Findings are interpreted as relative reporting, not incidence or absolute risk.

Analysis	Outcome/Comparison	Effect Estimate	95% CI	Interpretation
Adjusted logistic regression	CAR-T exposure → SKIN_ANY	aOR 0.13	0.09–0.20	Reduced reporting
Adjusted logistic regression	CAR-T exposure → broad SCAR	aOR 0.35	0.23–0.52	Reduced reporting
Adjusted logistic regression	Lymphodepletion → broad SCAR	aOR 0.54	0.48–0.61	Reduced reporting
Adjusted logistic regression	CRS co-mention → broad SCAR	aOR 3.70	3.21–4.26	Increased reporting
Within CAR-T model	CRS co-mention → SKIN_ANY	aOR 1.65	1.34–2.02	Increased reporting
Product-level disproportionality	Tisagenlecleucel → vascular cutaneous	ROR 1.81	1.05–3.12	Exploratory; attenuated by infection/cytopenia proxies

aOR = adjusted odds ratio; ROR = reporting odds ratio; CI = confidence interval; DLBCL = diffuse large B-cell lymphoma.

**Figure 2:**
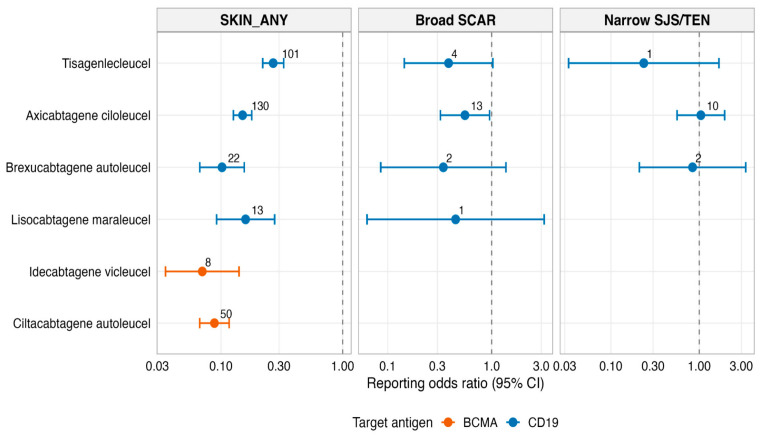
Per-product disproportionality across primary dermatologic outcomes among reports listing a CAR T-cell product as the primary suspect agent. Points show reporting odds ratios with 95% confidence intervals from product-level disproportionality analyses against the all-FAERS comparator. Numbers near points indicate exposed event counts; colours indicate target antigen. Obecabtagene autoleucel was omitted owing to insufficient cases. Product-by-category multiplicity assessment is provided in Supplementary Table S13.

In the primary-suspect product-level analysis, product totals summed to 16,888 reports. This analysis supported a class-wide pattern of reduced reporting across six evaluable products. SKIN_ANY RORs were significantly below unity for idecabtagene vicleucel (0.07, 95% CI 0.03–0.14), ciltacabtagene autoleucel (0.09, 95% CI 0.07–0.12), brexucabtagene autoleucel (0.10, 95% CI 0.07–0.16), axicabtagene ciloleucel (0.15, 95% CI 0.13–0.18), lisocabtagene maraleucel (0.16, 95% CI 0.09–0.28) and tisagenlecleucel (0.27, 95% CI 0.22–0.33) (Figure 2). Obecabtagene autoleucel had insufficient cases for interpretation. The only elevated product-by-category signal retained after full-family Benjamini–Hochberg correction was tisagenlecleucel with vascular cutaneous events (ROR 1.81, 95% CI 1.05–3.12; 13 cases; q = 0.046 across the 7 × 14 product-by-category family, 56 estimable cells). However, this signal was marginal and attenuated after excluding infection-attributable skin events and cytopenia/bleeding proxy reports, supporting an exploratory interpretation of bleeding/cytopenia co-reporting rather than a confirmed independent product-specific immune-mediated toxicity (Table 3 and Tables S11–S13).

Within CAR-T-exposed reports, indication-stratified findings should be interpreted cautiously. After a strict dictionary audit, the CAR-T × Hodgkin lymphoma subgroup comprised only 13 reports and no SKIN_ANY events, so the Hodgkin-versus-DLBCL contrast was not estimable, and the previously explored Hodgkin signal was not retained. Population-level Hodgkin lymphoma reports showed lower SKIN_ANY reporting (ROR 0.79, 95% CI 0.73–0.86). Multiple myeloma was not associated with increased dermatologic reporting after adjustment (aOR 0.96, 95% CI 0.53–1.74), and mantle cell lymphoma showed crude SCAR excess that attenuated after adjustment. CRS co-mention independently predicted SKIN_ANY within the CAR-T cohort (aOR 1.65, 95% CI 1.34–2.02; Table 4).

**Figure 3:**
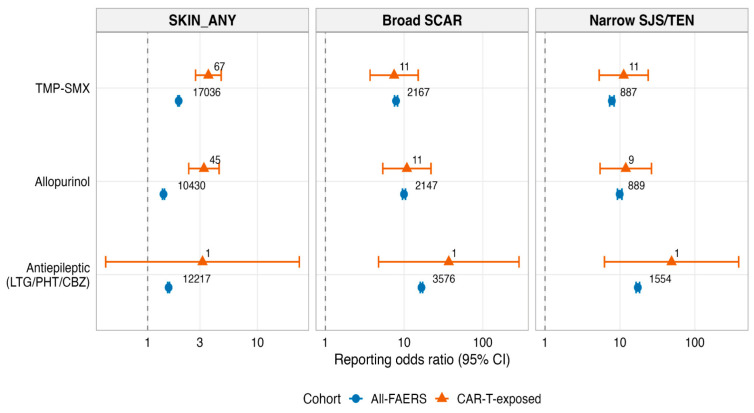
Strong-culprit signals in all-FAERS versus CAR-T-exposed reports. Points show reporting odds ratios with 95% confidence intervals for selected high-risk SJS/TEN culprit drugs across SKIN_ANY, broad SCAR and narrow SJS/TEN outcomes. Numbers near points indicate case counts. These analyses are descriptive and evaluate reporting patterns rather than causal drug-drug interactions. TMP-SMX = trimethoprim-sulfamethoxazole; LTG = lamotrigine; PHT = phenytoin; CBZ = carbamazepine.

Classical SCAR culprit drugs retained strong positive signals in the wider FAERS population. Individual culprit-drug reporting patterns are shown in Figure 3. Additive interaction analyses supported sub-additive joint effects for CAR-T with strong culprit drugs at the population level. Within CAR-T-exposed reports, concurrent trimethoprim-sulfamethoxazole and allopurinol exposure identified a small but clinically relevant high-risk subgroup, with substantially elevated narrow SJS/TEN reporting compared with other CAR-T-exposed reports (Supplementary Table S5).

**Figure 4:**
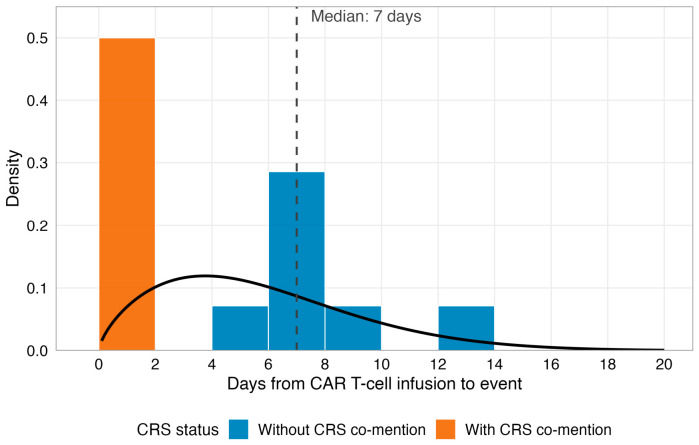
Time-to-onset distribution for CAR-T-associated narrow SJS/TEN events with Weibull fit overlay. The histogram is restricted to date-complete reports with valid therapy-start and event dates; it characterises timing among reported events and does not estimate incidence. The dashed line indicates the median onset of 7 days. All 10 date-complete events occurred within 14 days of infusion. The fitted Weibull distribution had shape = 1.67 and scale = 6.50. Colour indicates CRS co-mention. CRS = cytokine release syndrome; SJS/TEN = Stevens-Johnson syndrome/toxic epidermal necrolysis.

For the narrow SJS/TEN sensitivity outcome, 12,310 reports were identified overall, including 29 CAR-T-associated cases. The adjusted odds ratio for CAR T-cell exposure was not increased (aOR 0.84, 95% CI 0.51–1.37). Among 10 date-complete CAR-T-associated narrow SJS/TEN reports, the median time-to-onset was 7 days, and all occurred within 14 days of infusion. Among all 29 CAR-T-associated SJS/TEN reports, 16 (55.2%) co-mentioned CRS (Figure 4).

Clinical characterisation of severe dermatologic reports showed substantial seriousness and frequent co-reporting of CRS and supportive-care medications. Among CAR-T-associated broad SCAR reports, death, hospitalisation and life-threatening outcomes were reported in 18.4%, 65.8% and 26.3%, respectively; 57.9% co-mentioned CRS and 34.2% co-mentioned a strong SJS/TEN culprit drug. Vascular cutaneous reports had higher co-reporting of infection and cytopenia/bleeding proxies (38.9% and 58.3%, respectively), supporting cautious interpretation of vascular terms as potentially reflecting bleeding, infection or mixed supportive-care phenotypes rather than isolated immune-mediated skin toxicity (Supplementary Table S10).

Temporal analyses showed that the SKIN_ANY reduced-reporting signal was stable across calendar-year bands, with point estimates remaining below unity throughout the post-approval period. Exploratory Cox timing models for SKIN_ANY showed earlier recorded dermatologic reporting after CD19 and BCMA CAR T-cell exposure among reports with usable therapy dates (mechanism-based model: CD19 hazard ratio [HR] 2.34, 95% CI 1.98–2.77, *p* < 0.001; BCMA HR 2.58, 95% CI 1.34–4.97, *p* = 0.004; time-dependent model: CD19 HR 2.89, 95% CI 2.45–3.40, *p* < 0.001; BCMA HR 3.26, 95% CI 1.69–6.28, *p* < 0.001; Supplementary Table S7). These Cox analyses describe timing among reports with usable therapy dates and should not be interpreted as incidence estimates. 

Cross-database validation in Canada Vigilance reproduced the primary SKIN_ANY reduced-reporting signal despite a much smaller CAR-T numerator. In contrast, Canada Vigilance estimates for broad SCAR and narrow SJS/TEN were based on single-digit CAR-T-associated event counts and were therefore unstable. Although broad SCAR crossed the nominal signal threshold against the haematological-malignancy comparator, this estimate was based on only three CAR-T-associated events and should be considered exploratory rather than confirmatory (Supplementary Figures S3–S5).

Comparator-robustness analyses confirmed that the primary SKIN_ANY reduced-reporting pattern persisted across all-FAERS, haematological-malignancy indication and active haematology-oncology therapy comparators (ROR 0.17, 0.21 and 0.16, respectively). Broad SCAR remained directionally reduced but attenuated against the active haematology-oncology comparator, while narrow SJS/TEN was sparse and comparator-sensitive. Multiplicity analyses showed that 13 of 14 phenotype-specific categories remained significant after Benjamini–Hochberg correction; vascular cutaneous did not survive FDR correction at the class level. The tisagenlecleucel vascular cutaneous signal was the sole elevated survivor in the complete product-by-category testing family, but it was borderline (q = 0.046) and lost statistical significance after excluding infection-attributable skin events or cytopenia/bleeding proxy reports (Supplementary Tables S11–S13).

**Table 3:** Product-level SKIN_ANY disproportionality and notable findings among reports listing a CAR T-cell product as the primary suspect agent.

Product	Target	N Total	SKIN_ANY Cases	SKIN_ANY ROR (95% CI)	Notable Finding
Axicabtagene ciloleucel	CD19	6575	130	0.15 (0.13–0.18)	Broad SCAR 0.55 (0.32–0.95)
Tisagenlecleucel	CD19	2907	101	0.27 (0.22–0.33)	Vascular cutaneous 1.81 (1.05–3.12)
Ciltacabtagene autoleucel	BCMA	4274	50	0.09 (0.07–0.12)	Consistent low reporting
Brexucabtagene autoleucel	CD19	1632	22	0.10 (0.07–0.16)	No elevated signal
Lisocabtagene maraleucel	CD19	622	13	0.16 (0.09–0.28)	No elevated signal
Idecabtagene vicleucel	BCMA	859	8	0.07 (0.03–0.14)	No elevated signal
Obecabtagene autoleucel	CD19	19	<3	Insufficient	Not interpretable

ROR = reporting odds ratio; CI = confidence interval; SCAR = severe cutaneous adverse reactions. Product-level analyses were restricted to primary-suspect CAR T-cell reports to improve product attribution. Therefore, product totals and SKIN_ANY case counts differ from the broader any-role CAR-T cohort shown in Table 1.

**Table 4:** Within-CAR-T multivariable logistic regression for SKIN_ANY reporting: selected indication and clinical predictors.

Predictor	Adjusted OR	95% CI	*p*-Value	Interpretation
Follicular lymphoma vs. DLBCL	1.31	0.75–1.75	0.31	Not significant
Mantle cell lymphoma vs DLBCL	1.10	0.87–1.64	0.66	Crude excess attenuated
Multiple myeloma vs. DLBCL	0.96	0.53–1.74	0.90	Not increased
Acute lymphoblastic leukaemia vs. DLBCL	0.94	0.67–1.41	0.76	Not increased
CRS co-mention	1.65	1.34–2.02	<0.001	Independent within-cohort predictor
Polypharmacy, per drug	1.03	1.02–1.04	<0.001	Modest increase

The reference indication is diffuse large B-cell lymphoma. Model adjusted for target antigen, age, sex, polypharmacy, immune checkpoint inhibitor exposure, lymphodepleting chemotherapy, strong/weak culprit co-exposure and CRS co-mention. Strict Hodgkin lymphoma audit identified only 13 CAR-T × Hodgkin lymphoma reports with no SKIN_ANY events; this contrast was therefore not estimable and is not retained in the table.

## 4. Discussion

This decade-wide pharmacovigilance analysis found no evidence of increased reporting of dermatologic adverse events with CAR T-cell therapy at the class level. Instead, CAR T-cell exposure was associated with reduced reporting odds for the primary dermatologic outcome and for broad SCAR, with consistent reductions across phenotype-specific categories and across established products. The finding should not be interpreted as proof that CAR T-cell therapy prevents skin toxicity or reduces true clinical incidence. Rather, it indicates that within spontaneous reporting systems, dermatologic adverse events are reported less frequently with CAR T-cell therapy than with comparators, after accounting for major measured confounders. The revised sensitivity analyses further support this interpretation, showing that the primary SKIN_ANY reduced-reporting pattern persisted across comparator populations and was not materially altered by HSCT/GVHD co-reporting proxies. This interpretation is consistent with established CAR T-cell toxicity frameworks, in which CRS, ICANS, cytopenias, infection and supportive-care medication exposure dominate early post-infusion morbidity [17,18].

The pattern across phenotype-specific categories is biologically coherent but should be interpreted as relative reporting rather than clinical incidence. The strongest reductions occurred in psoriasiform, pruritic, eczematous, urticarial and hair-change categories, which are more likely to involve chronic inflammatory or immune-mediated pathways. By contrast, the weakest reductions occurred in acute drug eruption, skin lesion, and vascular categories, where events may reflect cytokine activation, thrombocytopenia, endothelial injury, infection, supportive care drugs or mixed mechanisms. The new infection/cytopenia analyses support this mixed-mechanism interpretation: vascular cutaneous reports showed substantial co-reporting of infection and cytopenia/bleeding proxies, and the tisagenlecleucel vascular signal attenuated after these proxies were excluded or adjusted for.

Infection and cytopenia/bleeding were therefore handled as competing clinical explanations and potential misclassification pathways, rather than as generic causal risk factors. Infectious complications after CD19-directed CAR T-cell therapy are well recognised, particularly in the context of lymphodepletion, cytopenias, B-cell aplasia and intensive supportive care [19,20]. Cutaneous infections, viral reactivation, wound infection, thrombocytopenic purpura, petechiae and ecchymoses can all enter spontaneous reporting systems as skin events, even though they would usually be clinically distinguishable through examination, blood counts, microbiology, imaging and treatment response. Sensitivity analyses that excluded infection-attributable terms and adjusted for cytopenia/bleeding proxies were used to test this alternative explanation.

These findings extend and partially reframe prior FAERS work. Storgard et al. reported elevated severe cutaneous eruptions and vascular cutaneous signals with selected CD19-directed products [6]. In our extended dataset, the broad class-level pattern showed reduced reporting, and the prior-study replication using the Storgard et al. [6] analytic frame suggested attenuation of early post-approval signals over longer follow-up. The tisagenlecleucel-specific vascular cutaneous signal persisted after full-family FDR correction only marginally and was the sole elevated survivor of the 56 estimable product-by-category cells. However, the signal was no longer significant after excluding infection-attributable skin events or cytopenia/bleeding proxy reports. This supports an exploratory interpretation most consistent with bleeding/cytopenia co-reporting and argues against presenting the signal as a definitive product-specific immune-mediated dermatologic toxicity.

The temporal analyses support a clinically important message: rare severe acute cutaneous events reported after CAR T-cell therapy cluster within the early CRS window. Date-complete CAR-T-associated SJS/TEN reports had a median onset of 7 days, and all occurred within 14 days. This timing is earlier than classical delayed drug-induced SJS/TEN, a severe drug-associated epidermal necrolysis syndrome with substantial morbidity and mortality [21], and supports careful distinction between cytokine-associated cutaneous toxicity and classical immune-mediated drug reactions. In practice, an early, severe rash after CAR T-cell therapy should prompt a coordinated assessment for CRS, infection, cytopenias, supportive care drugs, and true severe cutaneous adverse reactions.

Clinical implications are therefore focused on evaluation and attribution rather than reduced surveillance. In CAR T-cell recipients, a new severe rash or vascular cutaneous change should prompt parallel assessment for CRS timing, platelet count and other cytopenias, infection or viral reactivation, recent antimicrobial and allopurinol exposure, and true immune-mediated SCAR. The findings support a low threshold for dermatology input when morphology is severe, progressive, bullous, mucosal, necrotic, purpuric or diagnostically uncertain, but they do not support de-escalating routine monitoring for cutaneous toxicity after CAR T-cell therapy. This is consistent with contemporary EBMT/JACIE/EHA best-practice recommendations and the ASCO guideline on immune-related adverse events after CAR T-cell therapy, both of which emphasise structured monitoring, multidisciplinary toxicity management and supportive care for adults and children receiving CAR T-cell therapy [22,23].

The supportive-care and severe-event analyses add practical nuance. Severe cutaneous reports after CAR T-cell therapy were clinically heterogeneous, frequently co-mentioned CRS and often carried serious outcomes such as hospitalisation, life-threatening designation or death. Co-reported tocilizumab, corticosteroids, antibiotics and antivirals should be interpreted as contextual management proxies rather than proof of treatment for the dermatologic event. Within CAR-T-exposed reports, combined trimethoprim-sulfamethoxazole and allopurinol exposure identified a small subgroup with markedly higher SJS/TEN reporting. This is clinically relevant because antimicrobial prophylaxis and infection-prevention strategies are routine components of CAR T-cell supportive care, particularly in the setting of lymphodepletion, cytopenias and infection risk [24,25]. Haematologic toxicity after CD19 CAR T-cell therapy is also well recognised, including early and late cytopenias, and the CAR-HEMATOTOX model and subsequent immune effector cell-associated haematotoxicity framework further emphasise the clinical importance of post-CAR-T cytopenias for infection risk and supportive-care needs [26,27,28]. These data support our interpretation of cytopenia/bleeding proxies as clinically plausible contributors to purpuric, petechial, ecchymotic or vascular cutaneous reporting after CAR T-cell therapy. Endothelial activation, coagulopathy and endothelial injury syndromes have also been described after CAR T-cell therapy, providing a biologically plausible framework for interpreting selected vascular cutaneous reports as potentially mixed endothelial, inflammatory, bleeding or supportive-care phenotypes rather than isolated immune-mediated dermatologic toxicity [29,30]. The finding supports vigilance for severe rash or vascular skin changes in CAR T-cell recipients receiving high-risk supportive-care combinations, while recognising that FAERS cannot establish causality or treatment response.

Our cross-database, temporal and comparator analyses strengthen the central finding for the primary SKIN_ANY outcome. Reduced relative reporting was observed across all-FAERS, haematological-malignancy indication and active haematology-oncology therapy comparators, and Canada Vigilance provided directionally concordant primary-outcome validation despite a much smaller CAR-T numerator. In contrast, broad SCAR and narrow SJS/TEN were sparse and more comparator-sensitive; therefore, these severe-outcome analyses should be interpreted as exploratory and signal-generating rather than confirmatory risk estimates.

## 5. Limitations

This study has the inherent limitations of spontaneous reporting databases. FAERS and Canada Vigilance cannot estimate incidence, absolute risk or comparative clinical frequency because the exposed denominator is unknown and reporting is influenced by clinical severity, novelty, litigation, publicity and regulatory attention. Disproportionality analyses are designed to identify patterns of disproportionate reporting rather than causal effects or patient-level risks [31]. A reduced reporting odds ratio should therefore be interpreted as reduced relative reporting, not reduced true clinical incidence.

Clinical details are limited. Dermatology adjudication, rash morphology, biopsy findings, infection status, platelet counts, CRS grade, management and rechallenge data are unavailable or inconsistently reported. FAERS cannot reliably distinguish true immune-mediated dermatologic toxicity from infectious, bleeding/cytopenia-related, endothelial, treatment-related or supportive-care-related cutaneous manifestations. Some events coded as SJS/TEN may represent cytokine-driven mucocutaneous inflammation or severe exanthema, while some true cutaneous toxicities may be under-reported if low-grade or clinically overshadowed by CRS, ICANS, infection or cytopenias.

Residual confounding remains likely despite adjustment. CAR T-cell products are used in different diseases, age groups, treatment eras and supportive-care pathways. Prior autologous or allogeneic HSCT, chronic GVHD and transplant-related endothelial injury are clinically important potential confounders but are not reliably encoded in FAERS. We therefore performed a conservative HSCT/GVHD co-reporting proxy analysis, which showed no material effect on the main estimates; however, the absence of co-reporting cannot rule out undocumented transplant history. Indication coding in FAERS is incomplete, and product-level and indication-level outliers should therefore be interpreted as signal-generating rather than definitive evidence of differential product safety.

Finally, rare-event analyses are numerically fragile and vulnerable to multiple testing. Narrow SJS/TEN time-to-onset, Canada Vigilance SCAR estimates, and the tisagenlecleucel vascular cutaneous signal were based on small numbers and should be viewed as exploratory. Although the tisagenlecleucel vascular signal survived full-family Benjamini–Hochberg correction marginally, its attenuation after infection-attributable and cytopenia/bleeding proxy exclusions supports cautious interpretation. Confirmation requires prospective registries, clinical chart review, dermatology adjudication, validated transplant history, infection adjudication, platelet count data and linkage to treatment denominators.

## 6. Conclusions

Within spontaneous reporting systems, CAR T-cell therapy was not associated with increased relative reporting of dermatologic adverse events and instead showed reduced reporting odds across broad, phenotype-specific, product-level, temporal and externally replicated analyses. These findings should not be interpreted as reduced true incidence or clinical risk. Severe cutaneous outcomes were sparse and clinically heterogeneous, with frequent co-reporting of CRS, infection, cytopenia/bleeding proxies and supportive-care drugs. Clinically, early severe cutaneous events after infusion should be evaluated using a mechanism-aware approach that considers CRS, infection, cytopenias/bleeding, supportive care drugs, and true immune-mediated SCAR in parallel. Product- and indication-specific findings should be validated in prospective registries or chart-reviewed clinical cohorts.

## Data Availability

FAERS data are publicly available from the U.S. Food and Drug Administration. Canada Vigilance data are publicly available from Health Canada. Derived dictionaries, code and output tables can be made available on reasonable request, subject to repository or journal requirements.

## Supplementary Materials

The following supporting information can be downloaded at: https://www.mdpi.com/article/10.3390/cancers18132128/s1, Supplementary Methods: Extended dictionary, comparator, sensitivity and validation definitions; Supplementary Table S1: Mortality and case-fatality analyses; Supplementary Table S2: Replication of Storgard et al. using the original dictionary and extended follow-up; Supplementary Table S3: Temporal stability by calendar-year band; Supplementary Table S4: Sex- and age-stratified analyses; Supplementary Table S5: Strong-culprit drug-drug interaction analyses; Supplementary Table S6: Canada Vigilance external validation; Supplementary Table S7: Time-to-onset and Cox-model summaries; Supplementary Table S8: Cutaneous outcome-tier dictionary; Supplementary Table S9: HSCT/GVHD proxy confounding analyses. Supplementary Table S10: Clinical characterisation of severe dermatologic adverse events; Supplementary Table S11: Infection and cytopenia/bleeding sensitivity analyses; Supplementary Table S12: Comparator robustness analyses; Supplementary Table S13: Multiplicity and tisagenlecleucel vascular-signal credibility analyses; Supplementary Figures S1–S5: Prior-study replication, sensitivity analyses, cross-database validation, compact SKIN_ANY replication, and temporal-stability plots.
