# Supplementary material for: Real-World Dermatologic Adverse Events of CAR T-Cell Therapy: A Decade-Wide Disproportionality Analysis of the FDA Adverse Event Reporting System
Source: Cancers (Basel). 2026 Jun 30;18(13):2128. doi: 10.3390/cancers18132128 (PMC13359831; doi:10.3390/cancers18132128)
Supplement: Supplementary file 1 [file cancers-18-02128-s001.zip › cancers-4375996-supplementary.pdf]

Supplementary material

Real-world dermatologic adverse events of CAR T-cell therapy: a decade-wide disproportionality analysis of the FDA Adverse Event Reporting System

Supplementary material inventory

Supplementary Methods: Extended dictionary, comparator, sensitivity and validation definitions.
Supplementary Table S1: Mortality and case-fatality analyses.
Supplementary Table S2: Replication of Storgard et al. using the original dictionary and extended follow-up.
Supplementary Table S3: Temporal stability by calendar-year band.
Supplementary Table S4: Sex- and age-stratified analyses.
Supplementary Table S5: Strong-culprit drug-drug interaction analyses.
Supplementary Table S6: Canada Vigilance external validation.
Supplementary Table S7: Time-to-onset and Cox-model summaries.
Supplementary Table S8: Cutaneous outcome-tier dictionary.
Supplementary Table S9: HSCT/GVHD proxy confounding analyses.
Supplementary Table S10: Clinical characterisation of severe dermatologic adverse events.
Supplementary Table S11: Infection and cytopenia/bleeding sensitivity analyses.
Supplementary Table S12: Comparator robustness analyses.
Supplementary Table S13: Multiplicity and tisagenlecleucel vascular-signal credibility analyses.
Supplementary Figures S1-S5: Prior-study replication, sensitivity analyses, cross-database validation, compact SKIN_ANY replication, and temporal-stability plots.

Supplementary Methods:

FAERS tables were linked at the report level and deduplicated by retaining the most recent case version within composite keys. CAR T-cell exposure was identified using composite drug-name and active-ingredient regular expressions for CD19-directed and BCMA-directed products. Outcomes were defined using the MedDRA Skin and subcutaneous tissue disorders system organ class, a broad SCAR term set, a narrow SJS/TEN sensitivity definition and 14 phenotype-specific dermatologic categories adapted from Storgard et al.

Comparator analyses used both all-database and haematological-malignancy comparator populations. Product-level analyses were restricted to the primary-suspect CAR T-cell product role. Indication-stratified models were fitted within CAR-T-exposed reports, with DLBCL as the reference. Sensitivity layers excluded strong SJS/TEN culprit drugs, concomitant immune checkpoint inhibitors and events occurring within the first 14 days after CAR T-cell infusion.

Strong culprit drugs were lamotrigine, trimethoprim-sulfamethoxazole, phenytoin, allopurinol and carbamazepine. Weak culprit drugs were selected antimicrobial agents. Additive interaction was assessed using RERI, attributable proportion and synergy index. External validation used an equivalent Canada Vigilance time window and a Storgard-style replication cohort restricted to axicabtagene ciloleucel and tisagenlecleucel, with the original published dictionary applied before comparison with refined-dictionary results.

Additional sensitivity analyses evaluated HSCT/GVHD and transplant-associated endothelial injury using conservative co-reporting proxies; infection-attributable cutaneous terms; cytopenia, bleeding and coagulopathy proxies; severe dermatologic-event characteristics; comparator robustness; and multiplicity correction across the complete product-by-category testing family.

Supplementary Table S1: Mortality and case-fatality analyses

S1A: Overall mortality

cohort	N	N_deaths	mortality_pct
All FAERS	8431841	701113	8.3
CAR-T (any)	19200	4507	23.5
CAR-T x SKIN_ANY	425	54	12.7
CAR-T x broad SCAR	38	7	18.4
CAR-T x narrow SJS/TEN	29	6	20.7

S1B: Mortality by dermatologic category within CAR-T-exposed reports

category	N_cart_dae	N_deaths	mortality_pct
bullous_dermatosis	21	7	33.3
vascular_cutaneous	36	10	27.8
urticaria_angioedema	24	6	25
wound_ulcer	13	3	23.1
severe_cutaneous_eruption	28	6	21.4
skin_lesions	43	9	20.9
toxic_eruption	11	2	18.2

hyperhidrosis	24	4	16.7
pruritus	23	2	8.7
other_skin_changes	105	9	8.6
rash	129	8	6.2
hair_changes	23	1	4.3
eczematous	16	0	0
psoriasiform	2	0	0

Supplementary Table S2: Storgard et al (6) replication using the original dictionary

**category**	**storgard_pooled**	**storgard_tisa**	**storgard_axi**	**our_repl_pooled**	**our_repl_axi**	**our_repl_tisa**
bullous_dermatosis	0.40 (0.13-1.23)	0.32 (0.05-2.31)	0.45 (0.11-1.80)	0.32 (0.19-0.54)	0.28 (0.15-0.54)	0.42 (0.18-1.02)
eczematous	0.16 (0.07-0.33)	0.27 (0.11-0.65)	0.07 (0.02-0.30)	0.08 (0.04-0.15)	0.09 (0.04-0.19)	0.04 (0.01-0.25)
hair_changes	0.17 (0.08-0.38)	0.14 (0.04-0.56)	0.19 (0.07-0.52)	0.12 (0.07-0.22)	0.12 (0.06-0.25)	0.12 (0.04-0.39)
hyperhidrosis	0.87 (0.53-1.45)	0.71 (0.30-1.71)	0.99 (0.53-1.83)	0.27 (0.17-0.43)	0.22 (0.12-0.40)	0.41 (0.20-0.86)
other_skin_changes	0.89 (0.50-1.57)	1.82 (0.98-3.39)	0.25 (0.06-1.00)	0.14 (0.11-0.18)	0.12 (0.09-0.17)	0.20 (0.13-0.30)
pruritus	0.11 (0.05-0.27)	0.17 (0.05-0.51)	0.08 (0.02-0.30)	0.09 (0.06-0.13)	0.06 (0.04-0.11)	0.15 (0.08-0.28)
psoriasiform	0.12 (0.03-0.47)	<NA>	0.20 (0.05-0.80)	0.02 (0.01-0.10)	0.03 (0.01-0.13)	0.02 (0.00-0.35)
rash	0.43 (0.32-0.57)	0.61 (0.42-0.89)	0.31 (0.20-0.48)	0.19 (0.15-0.25)	0.16 (0.11-0.22)	0.29 (0.19-0.44)
severe_cutaneous_eruption	2.12 (0.53-8.47)	5.18 (1.29-20.76)	<NA>	0.40 (0.22-0.73)	0.45 (0.24-0.87)	0.27 (0.07-1.09)
skin_lesions	1.82 (0.91-3.63)	1.66 (0.54-5.17)	1.92 (0.80-4.61)	1.22 (0.83-1.79)	0.96 (0.58-1.60)	1.90 (1.05-3.44)
toxic_eruption	0.83 (0.21-3.32)	1.02 (0.14-7.21)	0.70 (0.10-4.98)	0.94 (0.35-2.51)	0.97 (0.31-3.00)	0.87 (0.12-6.16)

urticaria_angioedema	0.58 (0.34-0.98)	0.81 (0.41-1.63)	0.42 (0.19-0.94)	0.14 (0.08-0.24)	0.04 (0.01-0.13)	0.41 (0.22-0.73)
vascular_cutaneous	2.91 (1.51-5.60)	6.35 (3.17-12.73)	0.55 (0.08-3.87)	0.50 (0.36-0.70)	0.41 (0.27-0.63)	0.74 (0.44-1.25)
wound_ulcer	0.73 (0.27-1.95)	0.89 (0.22-3.58)	0.62 (0.15-2.47)	0.36 (0.22-0.57)	0.34 (0.20-0.61)	0.39 (0.16-0.93)

Supplementary Table S3: Temporal stability of dermatologic adverse event reporting by calendar-year band

Reporting odds ratios for CAR T-cell exposure across calendar-year bands using the FDA receipt date (fda_dt) anchor for temporal stratification, which provides 100% temporal coverage of the deduplicated cohort (n = 8,431,841). The 2016–2017 window contained no CAR T-cell-exposed reports for any of the three outcomes and is therefore not estimable. Subsequent calendar-year bands show a stable reduced reporting signal for SKIN_ANY (the primary outcome), with point estimates between 0.14 and 0.20 throughout the post-approval period. Broad SCAR estimates reached statistical significance in the 2021–2023 and 2024–2026 bands as event counts accrued. Narrow SJS/TEN estimates remained imprecise across all bands.

year_band	outcome	N_year	cart_in_year	events_cart	total_events	ROR	conf_low	conf_high	reduced_signal	signal_elevated
2016-2017	skin_any	5,479	0	0	278	—	—	—	NE	NE
2016-2017	scar_broad	5,479	0	0	33	—	—	—	NE	NE
2016-2017	sjs_ten_narrow	5,479	0	0	23	—	—	—	NE	NE
2018-2020	skin_any	1,374,545	1,722	53	185,100	0.204	0.155	0.268	TRUE	FALSE
2018-2020	scar_broad	1,374,545	1,722	3	5,356	0.446	0.144	1.384	FALSE	FALSE
2018-2020	sjs_ten_narrow	1,374,545	1,722	1	2,279	0.350	0.049	2.484	FALSE	FALSE
2021-2023	skin_any	4,357,630	7,825	172	460,001	0.190	0.163	0.221	TRUE	FALSE
2021-2023	scar_broad	4,357,630	7,825	12	13,964	0.477	0.271	0.841	TRUE	FALSE
2021-2023	sjs_ten_narrow	4,357,630	7,825	7	5,589	0.697	0.332	1.463	FALSE	FALSE
2024-2026	skin_any	2,694,187	9,653	200	351,275	0.141	0.122	0.162	TRUE	FALSE
2024-2026	scar_broad	2,694,187	9,653	23	10,718	0.597	0.396	0.899	TRUE	FALSE
2024-2026	sjs_ten_narrow	2,694,187	9,653	21	4,419	1.329	0.865	2.041	FALSE	FALSE

N_year = total deduplicated FAERS reports in the calendar-year band; cart_in_year = CAR T-cell-exposed reports in the band (any role); events_cart = CAR T-cell-associated outcome events in the band; total_events = outcome events in the full band cohort; ROR = reporting odds ratio with 95% confidence interval (conf_low, conf_high); reduced_signal = TRUE when 95% CI upper bound < 1; signal_elevated = TRUE when 95% CI lower bound > 1; NE = not estimable (zero CAR T-cell-exposed events in the band); SKIN_ANY = any cutaneous adverse event; SCAR = severe cutaneous adverse reactions; SJS/TEN = Stevens-Johnson syndrome / toxic epidermal necrolysis. Temporal anchor: FDA receipt date (fda_dt), which is populated for all deduplicated reports.

Supplementary Table S4: Sex- and age-stratified analyses

S4A: Sex-stratified RORs

sex	outcome	N_sex	cart_in_sex	events_cart	total_events	ROR	conf_low	conf_high	reduced_signal
Male	skin_any	2927036	9042	218	301244	0.215	0.188	0.246	TRUE
Male	scar_broad	2927036	9042	18	11559	0.502	0.316	0.798	TRUE
Male	sjs_ten_narrow	2927036	9042	16	4750	1.09	0.667	1.78	FALSE
Female	skin_any	4180506	5628	152	571420	0.175	0.149	0.206	TRUE
Female	scar_broad	4180506	5628	13	13785	0.7	0.406	1.21	FALSE
Female	sjs_ten_narrow	4180506	5628	7	5767	0.901	0.429	1.89	FALSE
Unknown	skin_any	1324299	4530	55	123990	0.119	0.091	0.155	TRUE
Unknown	scar_broad	1324299	4530	7	4727	0.431	0.205	0.906	TRUE
Unknown	sjs_ten_narrow	1324299	4530	6	1793	0.978	0.439	2.18	FALSE

S4B: Age-stratified RORs

age_stratum	outcome	N_stratum	cart_in_stratum	events_cart	total_events	ROR	conf_low	conf_high	reduced_signal	signal_elevated
Paediatric (<18y)	skin_any	347546	793	33	59904	0.208	0.147	0.295	TRUE	FALSE
Paediatric (<18y)	scar_broad	347546	793	1	2447	0.178	0.025	1.26	FALSE	FALSE
Paediatric (<18y)	vascular_cutaneous	347546	793	1	1655	0.263	0.037	1.87	FALSE	FALSE
Adult (18-64y)	skin_any	2504204	5850	169	364704	0.174	0.149	0.203	TRUE	FALSE
Adult (18-	scar_broad	2504204	5850	17	12265	0.59	0.367	0.953	TRUE	FALSE

64y)						2				
Adult (18-64y)	vascular_cutaneous	2504204	5850	18	7531	1.02	0.644	1.63	FALSE	FALSE
Older adult (>=65y)	skin_any	1718729	4967	98	184528	0.167	0.137	0.204	TRUE	FALSE
Older adult (>=65y)	scar_broad	1718729	4967	12	8785	0.471	0.267	0.83	TRUE	FALSE
Older adult (>=65y)	vascular_cutaneous	1718729	4967	12	5911	0.701	0.398	1.24	FALSE	FALSE

Supplementary Table S5: Strong-culprit interaction analyses

S5A: Within-CAR-T strong-culprit drug-pair interactions

Partner A	Partner B	Outcome	Joint N	Joint events	Joint ROR	Multiplicative interaction	RERI (95% CI)	AP	Synergy index	Signal
cul_TMP_SMX	cul_ALLOPURINOL	sjs_ten_narrow	272	9	33.74	10.9	30.89 (4.1 to 57.68)	0.915	17.66	TRUE
cul_TMP_SMX	cul_ALLOPURINOL	scar_broad	272	9	24.29	3.77	20.03 (1.47 to 38.59)	0.825	7.16	TRUE
cul_TMP_SMX	cul_ALLOPURINOL	skin_any	272	30	6.3	1.27	2.75 (0.027 to 5.48)	0.437	2.08	TRUE
cul_TMP_SMX	cul_ANTIEPILEPTIC	sjs_ten_narrow	4	1	336.5	0.717	281.4 (-502 to 1064.7)	0.836	6.2	FALSE
cul_TMP_SMX	cul_ANTIEPILEPTIC	scar_broad	4	1	224.2	1.08	187.8 (-331.8 to 707.4)	0.838	6.31	FALSE
cul_TMP_SMX	cul_ANTIEPILEPTIC	skin_any	4	1	16.6	2.08	11.81 (-26.34	0.711	4.11	FALSE

							to 49.95)			
cul_ALLOPURINOL	cul_ANTIEPILEPTIC	sjs_ten_narrow	4	1	307.8	0.687	256.1 (-459.3 to 971.5)	0.832	6.06	FALSE
cul_ALLOPURINOL	cul_ANTIEPILEPTIC	scar_broad	4	1	227.9	0.74	187.9 (-340.1 to 715.9)	0.825	5.82	FALSE
cul_ALLOPURINOL	cul_ANTIEPILEPTIC	skin_any	4	1	15.88	2.29	11.52 (-24.97 to 48.01)	0.725	4.43	FALSE

S5B: All-FAERS potentiation signals only

**Partner A**	**Partner B**	**Outcome**	**Joint N**	**Joint events**	**Joint ROR**	**Multiplicative interaction**	**RERI (95% CI)**	**AP**	**Synergy index**	**Signal**
cul_TMP_SMX	cul_ANTIEPILEPTIC	skin_any	1571	584	4.47	1.56	2.07 (1.61 to 2.53)	0.462	2.47	TRUE
cul_ALLOPURINOL	cul_ANTIEPILEPTIC	sjs_ten_narrow	948	67	63.43	0.331	35.78 (19.96 to 51.59)	0.564	2.34	TRUE
cul_ANTIEPILEPTIC	crs	scar_broad	331	76	94.67	1.11	74.01 (49.73 to 98.29)	0.782	4.76	TRUE
cul_ANTIEPILEPTIC	crs	skin_any	331	127	4.66	4.57	3.46 (2.42 to 4.49)	0.742	18	TRUE
cul_ANTIEPILEPTIC	lymphodepletion	sjs_ten_narrow	386	27	58.91	1.98	40.92 (17.85 to 63.99)	0.695	3.41	TRUE
cul_TMP_SMX	ici_concomitant	sjs_ten_narrow	1957	62	26.47	0.521	13.07	0.494	2.05	TRUE

							(6.34 to 19.81)			
cul_TMP_SMX	ici_concomitant	scar_broad	1957	119	20.41	0.676	9.59 (5.79 to 13.4)	0.47	1.98	TRUE

Supplementary Table S6: Canada Vigilance external validation

S6A: Cohort flow and demographics

**step**	**N**
Reports in Canada Vigilance (deduplicated, date-filtered)	743630
Any CAR T-cell product exposure	392
CD19 product exposure	391
BCMA product exposure	1
CAR-T x SKIN_ANY	4
CAR-T x broad SCAR	3
CAR-T x narrow SJS/TEN	1
characteristic	value
N	392
Median age (IQR)	61 (40-69)
Male (%)	59.2
Female (%)	34.7
Unknown sex (%)	6.1
Death outcome (%)	9.9

S6B: Class-level RORs

**outcome**	**comparator**	**a**	**ROR**	**conf_low**	**conf_high**	**signal_elevated**	**reduced_signal**
skin_any	All-Canada	4	0.07	0.026	0.186	FALSE	TRUE
skin_any	Haematological malignancy	4	0.15	0.056	0.403	FALSE	TRUE

scar_broad	All-Canada	3	2.57	0.826	8.02	FALSE	FALSE
scar_broad	Haematological malignancy	3	5.36	1.65	17.45	TRUE	FALSE
sjs_ten_narrow	All-Canada	1	2.41	0.339	17.19	FALSE	FALSE
sjs_ten_narrow	Haematological malignancy	1	10.97	1.32	91.34	FALSE	FALSE

Signal flags were not assigned to cells with fewer than three CAR-T-associated events, even when the continuity-corrected confidence interval crossed the nominal threshold; such estimates were considered numerically unstable and not interpreted as pharmacovigilance signals.

S6C: Fourteen-category panel in Canada Vigilance

category	cart_events	total_events	ROR	conf_low	conf_high	elevated	reduced
rash	0	26955	0.034	0.002	0.543	FALSE	FALSE
pruritus	0	22308	0.041	0.003	0.66	FALSE	FALSE
urticaria_angioedema	0	18633	0.05	0.003	0.794	FALSE	FALSE
psoriasiform	0	12311	0.076	0.005	1.21	FALSE	FALSE
hyperhidrosis	0	8732	0.107	0.007	1.72	FALSE	FALSE
other_skin_changes	2	33635	0.108	0.027	0.434	FALSE	FALSE
hair_changes	0	7635	0.123	0.008	1.97	FALSE	FALSE
skin_lesions	0	6400	0.147	0.009	2.35	FALSE	FALSE
bullous_dermatosis	0	4667	0.202	0.013	3.23	FALSE	FALSE
eczematous	0	4555	0.207	0.013	3.31	FALSE	FALSE
wound_ulcer	0	2901	0.326	0.02	5.21	FALSE	FALSE
vascular_cutaneous	1	2138	0.887	0.125	6.32	FALSE	FALSE
severe_cutaneous_eruption	1	1667	1.14	0.16	8.11	FALSE	FALSE
toxic_eruption	0	548	1.73	0.108	27.72	FALSE	FALSE

In Canada Vigilance category-level analyses, zero-event cells were retained for transparency using continuity-corrected estimates, but reduced/elevated signal flags were not assigned to zero-event cells because they were considered too sparse for stable interpretation.

Supplementary Table S7: Exploratory Cox-model summaries for SKIN_ANY timing analyses

Note: p-values are displayed as <0.001 where very small; values previously shown as 0 reflected spreadsheet or Word rounding, not a true p-value of zero.

S7A: Mechanism-based Cox model

Term	Hazard ratio	95% CI	p-value	Model
mechanismCD19	2.34	1.98-2.77	<0.001	mechanism_based_skin_any
mechanismBCMA	2.58	1.34-4.97	0.004	mechanism_based_skin_any

S7B: Time-dependent Cox model

Term	Hazard ratio	95% CI	p-value	Model
cd19_exp	2.89	2.45-3.40	<0.001	time_dependent_skin_any
bcma_exp	3.26	1.69-6.28	<0.001	time_dependent_skin_any

Supplementary Table S8: Cutaneous outcome-tier dictionary

Three nested cutaneous outcome tiers were used for the primary and sensitivity analyses.

Outcome tier	Operational definition / terms
**Any cutaneous AE (SKIN_ANY)**	Any MedDRA Preferred Term in the Skin and Subcutaneous Tissue Disorders System Organ Class; implemented in parallel with the 14 phenotype-specific dermatologic categories adapted from Storgard et al. [6], as described in the Supplementary Methods.
**SCAR-broad**	Selected SCAR-related MedDRA Preferred Terms, including Stevens-Johnson syndrome; toxic epidermal necrolysis; Stevens-Johnson syndrome/toxic epidermal necrolysis overlap; Lyell’s syndrome; toxic skin eruption; drug reaction with

	eosinophilia and systemic symptoms; erythema multiforme; exfoliative rash; exfoliative dermatitis; dermatitis exfoliative generalised; and acute generalised exanthematous pustulosis.
**SJS/TEN narrow**	Stevens-Johnson syndrome; Toxic epidermal necrolysis; Stevens-Johnson syndrome / toxic epidermal necrolysis overlap; Lyell’s syndrome; Toxic skin eruption.

Supplementary Figures

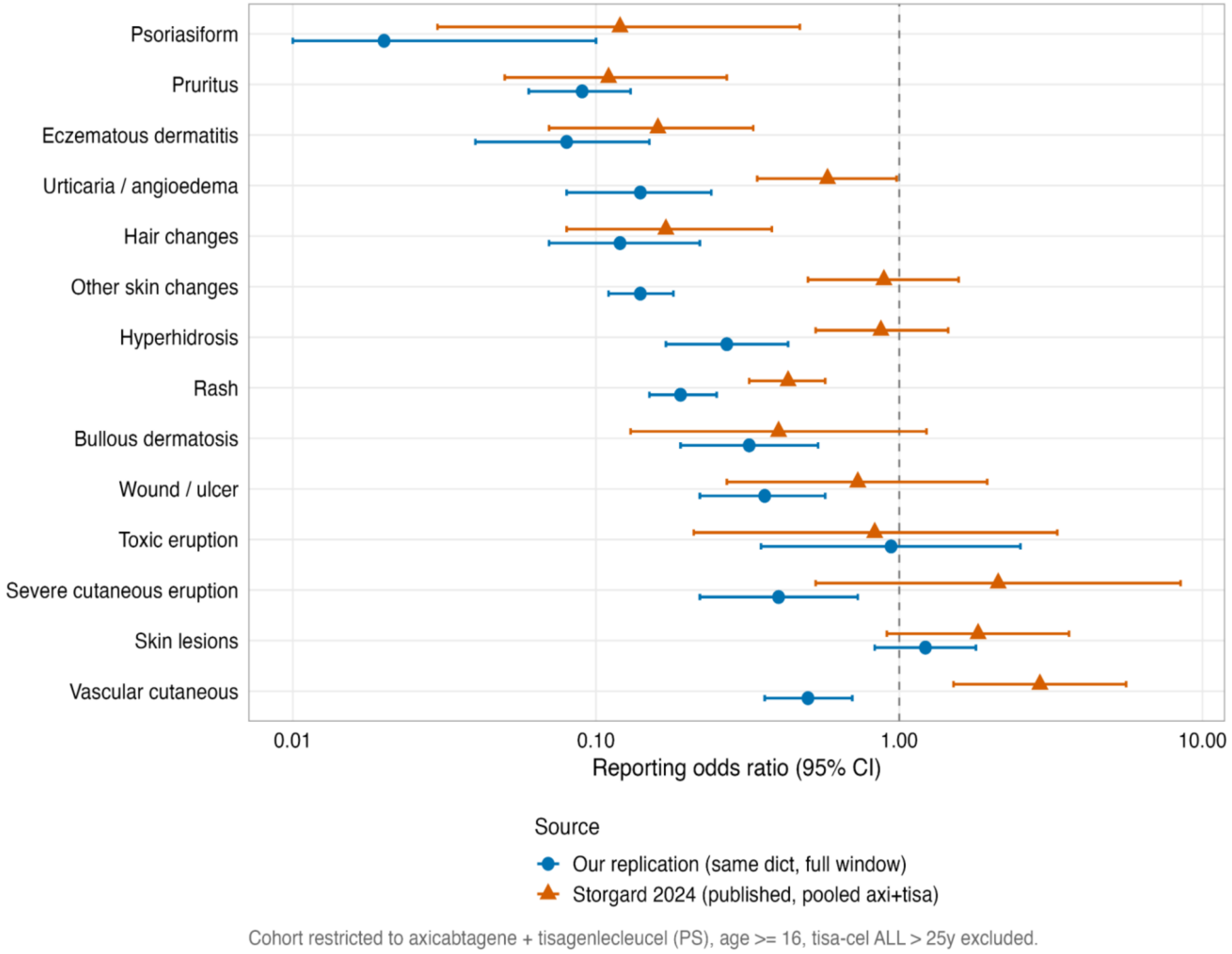

Supplementary Figure: S1 Prior-study replication of Storgard et al. using the original dictionary applied to an equivalent cohort with extended follow-up

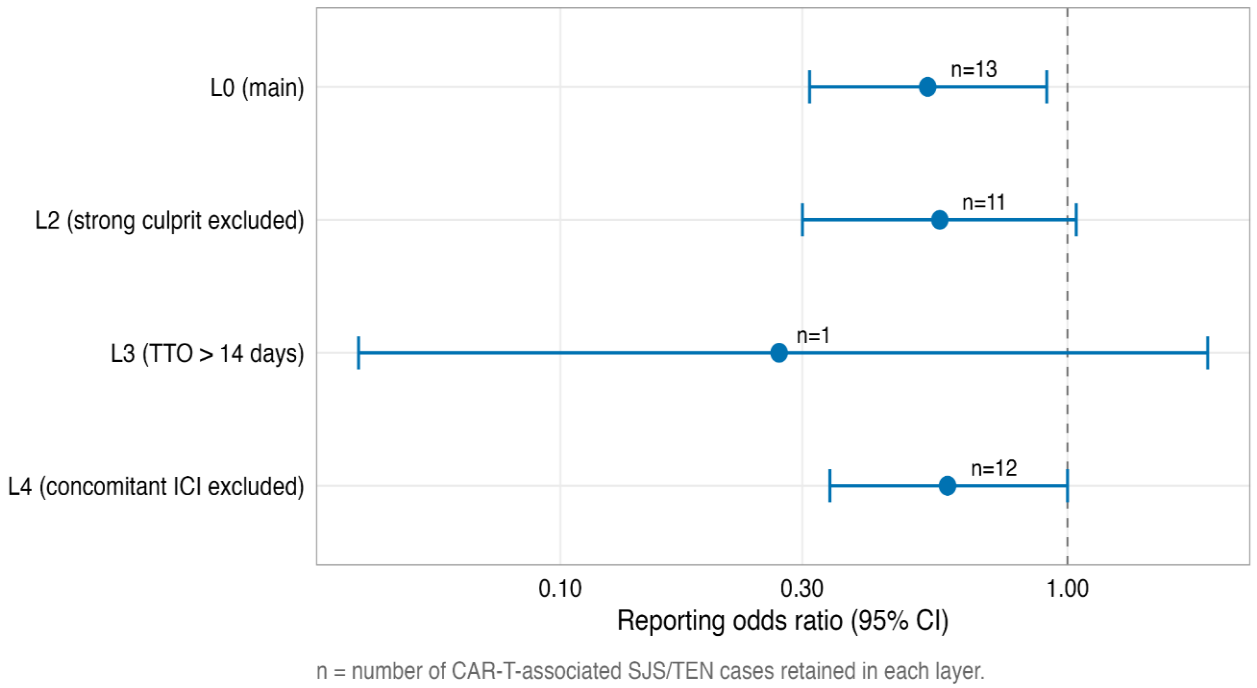

Supplementary Figure: S2 Sensitivity analyses of CAR-T-associated narrow SJS/TEN reporting odds ratios across prespecified sensitivity layers. n = number of retained CAR-T-associated SJS/TEN cases in each layer

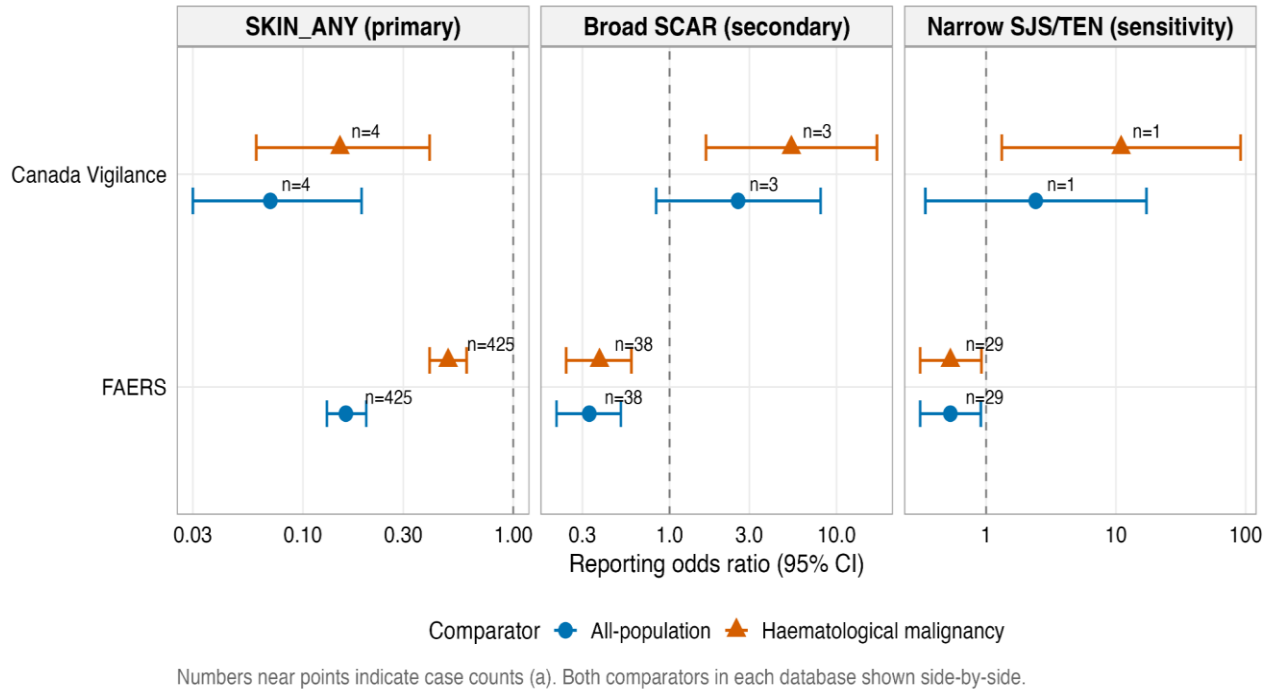

Supplementary Figure: S3 Cross-database external validation: FAERS versus Canada Vigilance across primary, secondary and sensitivity outcomes.

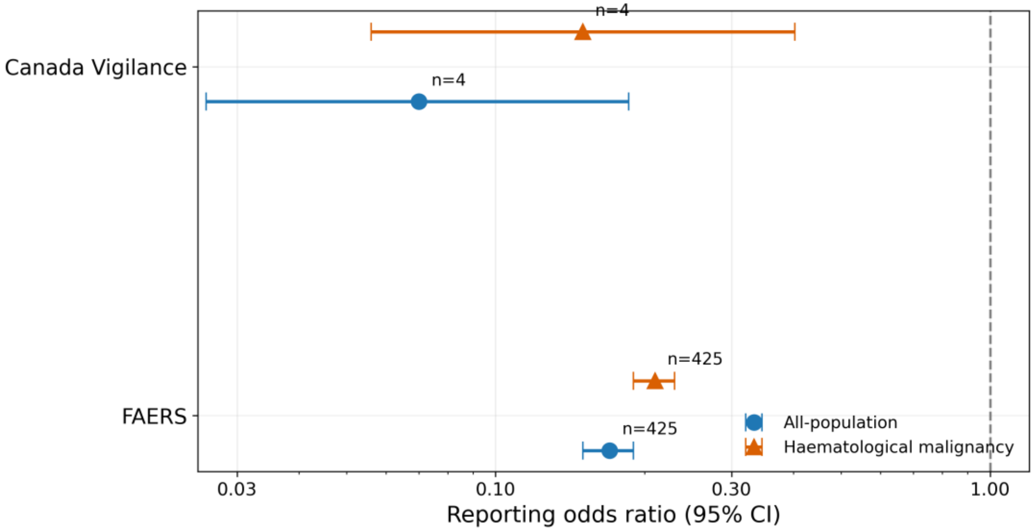

Reduced SKIN_ANY reporting is observed in both databases against both comparators.

Supplementary Figure: S4 Compact cross-database replication of reduced SKIN_ANY reporting with CAR T-cell therapy against all-population and haematological-malignancy comparators.

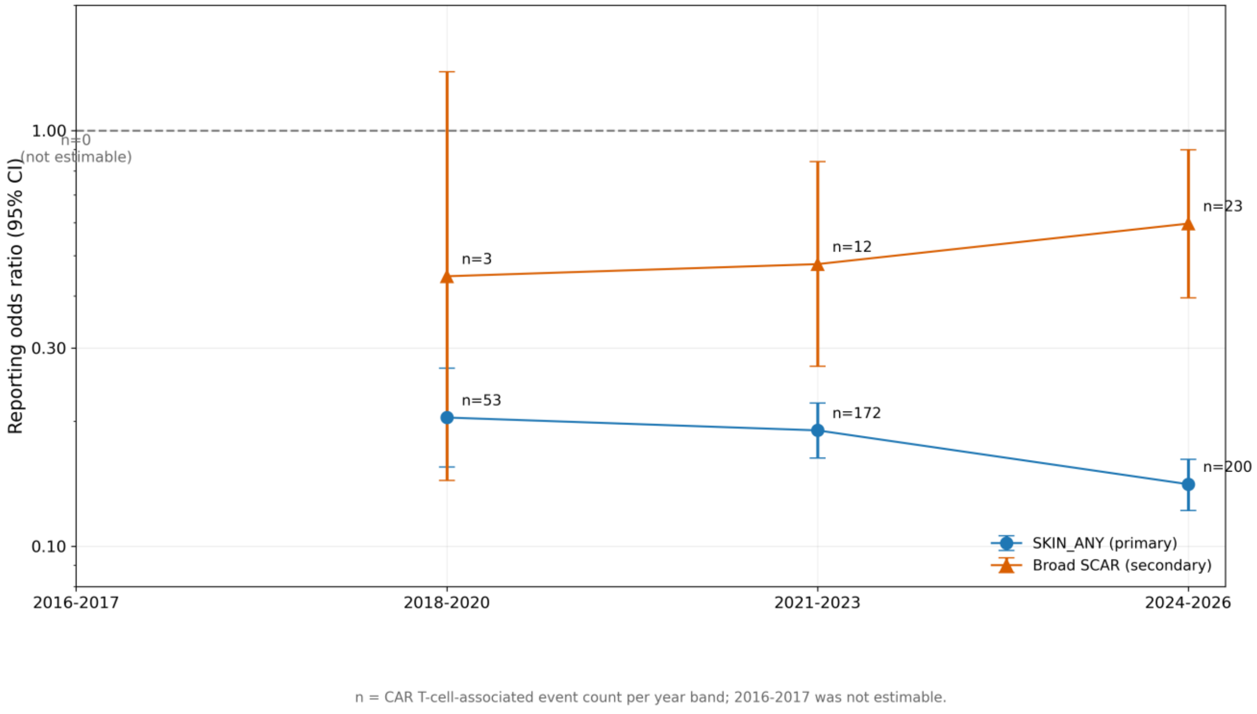

Supplementary Figure S5: Temporal stability of reduced dermatologic adverse-event reporting with CAR T-cell therapy across calendar-year bands. Numbers near points indicate CAR-T-associated event counts within each year band.

Supplementary Table S9: Prior haematopoietic stem-cell transplantation/graft-versus-host disease as a potential confounder

Prior HSCT and GVHD were ascertained as co-reporting proxies from MedDRA Preferred Terms (transplantation, graft-versus-host disease, and transplant-associated endothelial terms) and are a conservative floor for true prevalence, since prior transplant history is reported inconsistently in spontaneous reports.

S9A: HSCT/GVHD proxy frequency by CAR T-cell outcome stratum

Stratum	N	HSCT	GVHD	Endothelial	HSCT or GVHD	% HSCT/GVHD
CAR-T, any role	19,200	14	87	30	101	0.5
CAR-T × SKIN_ANY	425	0	9	3	9	2.1
CAR-T × broad SCAR	38	0	0	1	0	0.0
CAR-T × narrow SJS/TEN	29	0	0	0	0	0.0
CAR-T × vascular cutaneous	36	0	0	1	0	0.0
Tisagenlecleucel × vascular	13	0	0	0	0	0.0

S9B: CAR T-cell adjusted odds ratios before and after adding the HSCT/GVHD covariate

Note: These HSCT/GVHD sensitivity models used a compact adjustment set to minimise sparse-model instability and were intended to assess change after adding the HSCT/GVHD proxy, not to reproduce the primary multivariable estimates in Table 2.

Outcome	Base aOR (95% CI)	HSCT-adjusted aOR (95% CI)
SKIN_ANY	0.22 (0.19–0.25)	0.22 (0.19–0.25)
Broad SCAR	0.20 (0.14–0.30)	0.20 (0.14–0.30)
Vascular cutaneous	0.72 (0.49–1.06)	0.73 (0.50–1.07)

S9C: Tisagenlecleucel × vascular cutaneous, HSCT/GVHD exclusion sensitivity

Scope	N	Cases	ROR (95% CI)	p
All reports	8,431,841	13	1.81 (1.05–3.12)	0.033
Excluding HSCT/GVHD proxy	8,421,372	13	1.84 (1.07–3.17)	0.029

aOR = adjusted odds ratio; ROR = reporting odds ratio; CI = confidence interval; SCAR = severe cutaneous adverse reaction; SJS/TEN = Stevens-Johnson syndrome / toxic epidermal necrolysis. Adjusted models include age, sex, cytokine release syndrome, lymphodepleting chemotherapy, immune checkpoint inhibitor co-

exposure and strong-culprit co-exposure. The HSCT/GVHD proxy was absent from all broad SCAR, narrow SJS/TEN and vascular cutaneous reports, so adjustment leaves the CAR T-cell estimates essentially unchanged.

Supplementary Table S10: Clinical characterisation of severe dermatologic adverse events in CAR T-cell reports

Severe group	n	Death %	Hosp. %	Life-thr. %	CRS %	Infection %	Cytopenia %	Strong culprit %
Broad SCAR	38	18.4	65.8	26.3	57.9	10.5	21.1	34.2
Narrow SJS/TEN	29	20.7	62.1	20.7	55.2	6.9	20.7	37.9
Severe cutaneous eruption	28	21.4	75.0	17.9	60.7	7.1	10.7	35.7
Vascular cutaneous	36	27.8	33.3	8.3	66.7	38.9	58.3	22.2
Bullous dermatosis	21	33.3	61.9	19.0	57.1	23.8	23.8	28.6

Co-reported management agents (% of reports in each severe group)

Severe group	Tocilizumab %	Corticosteroid %	Antibiotic %	Antiviral %
Broad SCAR	21.1	21.1	47.4	31.6
Narrow SJS/TEN	20.7	20.7	62.1	37.9
Severe cutaneous eruption	21.4	21.4	53.6	42.9
Vascular cutaneous	5.6	5.6	27.8	38.9
Bullous dermatosis	23.8	4.8	38.1	28.6

S10B: Predictors of broad SCAR among CAR T-cell reports with any cutaneous AE (n = 425)

Predictor	SCAR among exposed	OR (95% CI)	p
Cytokine release syndrome	21	1.26 (0.63–2.51)	0.518
Infection (any)	3	0.34 (0.10–1.14)	0.080
Cytopenia (any)	6	0.37 (0.15–0.92)	0.032
Strong culprit co-exposure	13	2.62 (1.27–5.43)	0.009

CRS = cytokine release syndrome; OR = odds ratio; CI = confidence interval. Seriousness fields (death, hospitalisation, life-threatening) are derived from the FAERS outcome table. Management agents are co-reported medicinal products and indicate clinical context rather than confirmed treatment of the dermatologic event. Time

to onset is not tabulated per severe-event group because date-complete cases are too sparse for stable per-group medians; the validated time-to-onset finding (median 7 days for narrow SJS/TEN) is reported in the dedicated timing analysis. The HSCT/GVHD predictor was not estimable (zero exposed SCAR reports) and is omitted.

Supplementary Table S11: Infection- and cytopenia-attributable cutaneous events: sensitivity analyses

S11A. Reporting odds ratios excluding infection-attributable cutaneous events

Outcome	Full cases	Full ROR (95% CI)	Excl-infection cases	Excl-infection ROR (95% CI)
SKIN_ANY	425	0.17 (0.15–0.19)	384	0.16 (0.14–0.18)
Vascular cutaneous	36	0.76 (0.54–1.05)	32	0.70 (0.50–0.99)

S11B: Vascular cutaneous estimates adjusted for cytopenia and bleeding co-reporting

Analysis	Base aOR (95% CI)	Cytopenia/bleeding-adjusted aOR (95% CI)
CAR-T → vascular cutaneous	0.72 (0.49–1.06)	0.96 (0.64–1.46)
Tisagenlecleucel → vascular cutaneous	1.72 (0.94–3.17)	1.04 (0.54–2.02)

S11C. Infection and cytopenia proxy prevalence in the CAR T-cell cohort

Proxy	CAR-T reports (n)	CAR-T %
Skin infection	107	0.56
Viral cutaneous	365	1.90
Systemic infection	1,531	7.97
Thrombocytopenia	1,087	5.66
Neutropenia / pancytopenia	2,200	11.46
Bleeding / coagulopathy	444	2.31

aOR = adjusted odds ratio; ROR = reporting odds ratio; CI = confidence interval. The tisagenlecleucel × vascular cutaneous association attenuates to the null after adjustment for cytopenia and bleeding co-reporting and after exclusion of infection-attributable cutaneous events, consistent with a bleeding- or cytopenia-related phenotype rather than a distinct cutaneous toxicity.

Supplementary Table S12: Comparator robustness: CAR T-cell reporting odds ratios across three comparator populations

Outcome	A: all-FAERS	A cases	B: haem-onc indication	C: active haem-onc therapy
SKIN_ANY	0.17 (0.15–0.19)	425	0.21 (0.19–0.23)	0.16 (0.15–0.18)
Broad SCAR	0.55 (0.40–0.76)	38	0.57 (0.41–0.78)	0.77 (0.56–1.07)
Narrow SJS/TEN	1.03 (0.72–1.49)	29	0.94 (0.65–1.37)	1.49 (1.03–2.17)
Vascular cutaneous	0.76 (0.54–1.05)	36	0.67 (0.48–0.94)	0.54 (0.39–0.75)
Severe cutaneous eruption	0.48 (0.33–0.69)	28	0.62 (0.42–0.90)	0.78 (0.53–1.13)
Rash	0.22 (0.18–0.26)	129	0.14 (0.12–0.17)	0.11 (0.10–0.13)
Pruritus	0.06 (0.04–0.09)	23	0.08 (0.05–0.12)	0.05 (0.04–0.08)

Values are reporting odds ratios (95% CI) for CAR T-cell exposure versus each comparator. Comparator sizes: A, all non-CAR T-cell FAERS reports (n = 8,412,641); B, non-CAR T-cell reports with a haematological-malignancy indication (n = 491,180); C, non-CAR T-cell reports listing an active haematological-oncology therapy (n = 567,284). The reduced-reporting pattern is consistent across all three comparators. The nominally elevated narrow SJS/TEN estimate in comparator C is based on 29 exposed cases, is not consistent across comparators, and does not survive multiplicity adjustment.

Supplementary Table S13: Multiplicity (Benjamini-Hochberg FDR) and credibility of the tisagenlecleucel vascular signal

S13A: Class-level 14-category disproportionality with BH-FDR correction

Category	Cases	ROR	95% CI	q (BH)	Survives FDR
Other skin changes	105	0.13	0.11–0.16	<0.001	Yes
Rash	129	0.22	0.18–0.26	<0.001	Yes
Pruritus	23	0.06	0.04–0.09	<0.001	Yes
Urticaria / angioedema	24	0.11	0.07–0.17	<0.001	Yes
Eczematous	16	0.07	0.04–0.12	<0.001	Yes
Hair changes	23	0.12	0.08–0.18	<0.001	Yes
Hyperhidrosis	24	0.17	0.12–0.26	<0.001	Yes
Bullous dermatosis	21	0.23	0.15–0.35	<0.001	Yes
Psoriasiform	2	0.01	0.004–0.06	<0.001	Yes
Wound / ulcer	13	0.30	0.17–0.51	<0.001	Yes
Severe cutaneous eruption	28	0.48	0.33–0.70	<0.001	Yes

Skin lesions	43	0.61	0.45–0.83	0.002	Yes
Toxic eruption	11	0.44	0.24–0.79	0.006	Yes
Vascular cutaneous	36	0.76	0.55–1.05	0.093	No

S13B: Product × outcome disproportionality with BH-FDR correction (selected rows)

Note: Cells with fewer than three exposed events are shown for transparency but were not interpreted as stable pharmacovigilance signals.

Product	Outcome	Cases	ROR	95% CI	q (BH)	Survives
Tisagenlecleucel	SKIN_ANY	101	0.27	0.22–0.33	<0.001	Yes
Tisagenlecleucel	Broad SCAR	4	0.39	0.14–1.03	0.073	No
Tisagenlecleucel	Vascular cutaneous	13	1.81	1.05–3.12	0.046	Yes
Axicabtagene	Vascular cutaneous	17	1.04	0.65–1.68	0.862	No
Ciltacabtagene	Vascular cutaneous	1	0.09	0.01–0.67	0.034	Yes

S13C: Credibility audit of the tisagenlecleucel × vascular cutaneous signal

Analysis	Cases	Estimate	p
Primary (nominal ROR)	13	1.81 (1.05–3.12)	0.033
BH-FDR q (full 7×14 product×category family)	13	q = 0.046 (survives marginally; sole elevated survivor of 56 estimable)	0.046
Excluding HSCT/GVHD proxy	13	1.84 (1.07–3.17)	0.029
Excluding infection-attributable skin	10	1.46 (0.78–2.71)	0.234
Excluding cytopenia proxy	7	1.40 (0.67–2.94)	0.376

ROR = reporting odds ratio; CI = confidence interval; BH-FDR = Benjamini-Hochberg false-discovery-rate q-value; HSCT = haematopoietic stem-cell transplantation; GVHD = graft-versus-host disease. Thirteen of fourteen dermatologic categories show statistically reduced reporting after FDR correction. The tisagenlecleucel × vascular cutaneous association is the only elevated cell to survive FDR across the full pre-specified product-by-category family (7 products × 14 categories; 56 estimable cells; q = 0.046), and it does so marginally; it loses statistical significance when infection-attributable or cytopenia-related reports are excluded, indicating an exploratory, non-robust signal most consistent with bleeding/cytopenia co-reporting. Ciltacabtagene autoleucel showed reduced vascular cutaneous reporting, underscoring that the tisagenlecleucel finding is product-specific rather than class-wide.
